# Machine Learning for Outcome Prediction in First-Line Surgery of Prolactinomas

**DOI:** 10.3389/fendo.2022.810219

**Published:** 2022-02-16

**Authors:** Markus Huber, Markus M. Luedi, Gerrit A. Schubert, Christian Musahl, Angelo Tortora, Janine Frey, Jürgen Beck, Luigi Mariani, Emanuel Christ, Lukas Andereggen

**Affiliations:** ^1^ Department of Anaesthesiology and Pain Medicine, Inselspital, Bern University Hospital, University of Bern, Bern, Switzerland; ^2^ Department of Neurosurgery, Kantonsspital Aarau, Aarau, Switzerland; ^3^ Department of Gynecology and Obstetrics, Kantonsspital Lucerne, Lucerne, Switzerland; ^4^ Department of Neurosurgery, Inselspital, Bern University Hospital, University of Bern, Bern, Switzerland; ^5^ Department of Neurosurgery, Medical Center, University of Freiburg, Freiburg, Germany; ^6^ Department of Neurosurgery, University Hospital of Basel, Basel, Switzerland; ^7^ Department of Endocrinology, Diabetes and Metabolism, University Hospital of Basel, Basel, Switzerland; ^8^ Faculty of Medicine, University of Bern, Bern, Switzerland

**Keywords:** dopamine agonists, long-term outcome, machine learning, primary surgical therapy, prolactinoma, prediction modeling

## Abstract

**Background:**

First-line surgery for prolactinomas has gained increasing acceptance, but the indication still remains controversial. Thus, accurate prediction of unfavorable outcomes after upfront surgery in prolactinoma patients is critical for the triage of therapy and for interdisciplinary decision-making.

**Objective:**

To evaluate whether contemporary machine learning (ML) methods can facilitate this crucial prediction task in a large cohort of prolactinoma patients with first-line surgery, we investigated the performance of various classes of supervised classification algorithms. The primary endpoint was ML-applied risk prediction of long-term dopamine agonist (DA) dependency. The secondary outcome was the prediction of the early and long-term control of hyperprolactinemia.

**Methods:**

By jointly examining two independent performance metrics – the area under the receiver operating characteristic (AUROC) and the Matthews correlation coefficient (MCC) – in combination with a stacked *super learner*, we present a novel perspective on how to assess and compare the discrimination capacity of a set of binary classifiers.

**Results:**

We demonstrate that for upfront surgery in prolactinoma patients there are not a *one-algorithm-fits-all* solution in outcome prediction: different algorithms perform best for different time points and different outcomes parameters. In addition, ML classifiers outperform logistic regression in both performance metrics in our cohort when predicting the primary outcome at long-term follow-up and secondary outcome at early follow-up, thus provide an added benefit in risk prediction modeling. In such a setting, the stacking framework of combining the predictions of individual *base learners* in a so-called *super learner* offers great potential: the *super learner* exhibits very good prediction skill for the primary outcome (AUROC: mean 0.9, 95% CI: 0.92 – 1.00; MCC: 0.85, 95% CI: 0.60 – 1.00). In contrast, predicting control of hyperprolactinemia is challenging, in particular in terms of early follow-up (AUROC: 0.69, 95% CI: 0.50 – 0.83) vs. long-term follow-up (AUROC: 0.80, 95% CI: 0.58 – 0.97). It is of clinical importance that baseline prolactin levels are by far the most important outcome predictor at early follow-up, whereas remissions at 30 days dominate the ML prediction skill for DA-dependency over the long-term.

**Conclusions:**

This study highlights the performance benefits of combining a diverse set of classification algorithms to predict the outcome of first-line surgery in prolactinoma patients. We demonstrate the added benefit of considering two performance metrics jointly to assess the discrimination capacity of a diverse set of classifiers.

## Introduction

Dopamine agonists (DAs) are the treatment of choice for prolactinomas, given their effectiveness in controlling hyperprolactinemia and restoring gonadal function (1–3). However, in contrast to previous reports, most patients with low remission rates will need prolonged treatment with DAs (4). Additionally, potential long-time effects (5, 6), - including personality changes (7–10) - contributed to the increased acceptance of first-line surgery in prolactinomas in recent years (11–15). Although upfront surgery has recently been given a more dominant role in the treatment of prolactinomas (16, 17), their indication still remains controversial in selected patients (18, 19). Thus, accurate prediction of unfavorable outcomes after upfront surgery in prolactinoma patients is crucial to the triage of therapy and interdisciplinary decision-making. In this context of medical prognosis and prediction analysis, combining patient data with statistical methods, algorithms and tools that constitute the field of Machine Learning (ML) entails a distinct impact on medical research and clinical practice (20–25). As such, we aimed at examining whether and how contemporary ML methods can facilitate outcome prediction of first-line surgery in prolactinoma patients. In addition, we aimed at investigating the performance of various classes of supervised classification algorithms in predicting the risk of dependence on DAs over the long-term, as well as the control of hyperprolactinemia at early and long-term follow-up.

In particular, instead of finding a single best-performing model determined by a single performance metric, such as the commonly employed area under the receiver operating characteristic (AUROC), we aimed at focusing on quantifying and illustrating similarities and differences of the various classifiers by investigating two performance metrics jointly for our set of classifiers. We further aimed at providing a statistical framework to examine the cases for which ML methods offer an added benefit compared to traditional statistical approaches such as logistic regression. We will argue that by considering and combining multiple ML classifiers on the one hand and by examining two performance metrics jointly on the other hand, the utility of a set of patient- and treatment-related characteristics in predicting dependence on DAs and the risk of persistent hyperprolactinemia can be robustly investigated.

## Methods

### Study Design and Preoperative Assessment

This cohort study analyzed data from prolactinoma patients stored in our institutional database and prospectively maintained from January 1996 to December 2015. The Human Research Ethics Committee of Bern (Cantonal Ethikkommission KEK Bern, Bern, Switzerland) approved the project (KEK n° 10-10-2006 and 8-11-2006). Collected data included all consecutive prolactinoma patients with performance of upfront surgery in the treatment of either a micro- or macroprolactinoma. Thereby, a tumor diameter of 1–10 mm was characterized as a microadenoma and >10 mm as a macroadenoma, respectively. Invasiveness of the cavernous sinus was defined as Knosp grading ≥1 (11, 26, 27). Diagnosis of prolactinoma was based on biochemical and clinical assessment as well as on a standard protocol for the detection of pituitary adenomas with magnetic resonance imaging (MRI) (28–30). Biochemical measurements of PRL levels including the immunoradiometric PRL assay to overcome the high-dose PRL hook effect were completed (31), and the presence of macroprolactin was examined (32). Upper limits of >20 ng/mL were defined as hyperprolactinemia (33). Diagnosis was extended to immunohistochemical confirmation with a PRL antibody as an immunohistochemical marker according to the WHO classification of neuroendocrine tumors (34).

Partial hypopituitarism was considered when there was impaired secretion of one or more pituitary hormones. Secondary hypocorticism was defined in the presence of low serum cortisol (<50 nmol/L), or normal cortisol but inadequate responses to the insulin tolerance test or the adrenocorticotropin (ACTH) stimulation test. Secondary hypothyroidism was characterized by the presence of low-normal thyroid-stimulating hormone (TSH) levels along with a low free thyroxin (FT4) level. Central hypogonadism was defined as low-normal levels of gonadotropins in parallel with low estradiol/testosterone levels.

The indication for surgery was discussed by an interdisciplinary group at the weekly pituitary tumor board meeting, with consensus tailored to preventing patients from becoming dependent on DA therapy over the long term. The treatment decision was again discussed with the patient and the choice was based on his or her preference. Patients who had previously received DAs were excluded from the study.

### Postoperative and Long-Term Assessment

Early (short-term) follow-up occurred three months following surgery. If serum PRL levels were > 20 µg/L at that time, DA therapy was initiated (35), except in patients with prolactin levels slightly above the normal range but lacking clinical symptoms. In these patients, prolactin levels were subsequently reassessed. Late (Long-term) follow-up was defined as the last documented visit to the endocrine outpatient clinic. After initiation of DAs, medical therapy was tapered at 24 months if PRL levels were in the normal range (36, 37). Serum PRL level < 20 µg/L at last follow-up was characterized as in remission.

### Primary and Secondary Endpoints

The primary outcome is defined as long-term dependence on DAs. The secondary outcomes are defined as the successful control of hyperprolactinemia on early-term and long-term follow-up.

## Statistical Analysis and Prediction Modeling

### Descriptive Statistics and Predictors

In terms of descriptive statistics, continuous variables were examined with the Shapiro-Wilk normality test and are presented with mean and standard deviation for normally distributed variables and with median and interquartile range (IQR) otherwise. Categorical variables are presented with counts and percentages.

The following patients and treatment-related characteristics were available as predictors: age (numerical), sex (binary), adenoma size (binary, i.e. micro- vs. macro-adenoma), the incidence of headache at patients’ presentation (binary), partial hypopituitarism (binary), cavernous sinus invasion (binary), baseline prolactin levels (numerical) and remission at 30 days (binary; only used as a predictor of the long-term outcomes).

### Machine Learning Algorithms and Hyperparameter Selection

The selection of ML algorithms (the corresponding *R* packages are listed in italics) features a broad spectrum of algorithmic diversity and includes decision-tree-based algorithms [Random Forest, *randomForest* (38)], a distance-based algorithm [k-Nearest Neighbor, *kknn* (39)], standard (Logistic Regression) and penalized regression-based algorithms [Elasticnet Regularization; *glmnet* (40)], a feed-forward neural network with a single hidden layer [*nnet* (41)], flexible discriminant analysis [*earth* (42)], support vector machines [*e1071* (43)] as well as gradient boosting machines [*gbm* (44)]. A detailed description of each algorithm is beyond the scope of the present study and we refer the reader to the pertinent literature, e.g. (45, 46).

We adopted a heuristic approach to examine which algorithm-dependent hyperparameters are necessary to optimize in our setting. For each ML algorithm, we examined all hyperparameters and selected only those which (i) were tunable and (ii) featured a default value. For categorical hyperparameters, we sampled all possible predefined values uniformly. In case of integer or continuous hyperparameters, we sampled randomly and uniformly from an order of magnitude lower than the default value up to an order of magnitude greater than the default value (where numerically possible), thus accounting for the skewed nature of most continuous hyperparameters. For example, the default number of decision trees (*ntree*) in the Random Forest algorithm was set to 50, and we sampled accordingly from 5 to 500 trees. The importance of each hyperparameter was assessed by randomly sampling 50 values and examining the area under the curve (AUROC) in a three-fold repeated cross-validation sampling (RepCV) with 4 repetitions. Based on the AUROC distribution of each hyperparameter, we chose two hyperparameters for each algorithm. These were subsequently co-sampled. In addition to computing the performance of individual classifiers (so-called *base learners*), we combined the predictions of the base learners in a stacking framework in to a so-called *super learner* (47). We chose a gradient boosting machine as the *super learner*.

### Cross-Validation and Missing Data

A three-fold RepCV sampling with 100 repetitions was computed for each classifier and each outcome (the so-called *inner loop*), which was repeated for 100 different, randomly sampled hyperparameters combinations of each algorithm (the so-called *outer loop*).

The dataset features missing data at random in several variables, and data availability is indicated in each **Table 1**. Patients with missing data in the outcome variables are omitted in the prediction modeling (complete-case analysis). A single imputation method was used for missing predictor values: missing numerical data were imputed using the median value across the available patients, whereas the mode value was used for missing categorical variables. The single imputed dataset was used in the RepCV sampling.

**Table 1:** Patients’ characteristics at diagnosis.

Characteristics	All patients *(N*=86)
Age at diagnosis (years; *N*=85)	32.0 [27.0;42.0]
BMI (kg/m^2^; *N*=86)	26.4 (5.59)
Sex (female; *N*=86)	71 (82.6%)
Macroadenoma (*N*=69)	41 (47.7%)
Secondary hypogonadism (*N*=80)	53 (76.8%)
Secondary hypothyroidism (*N*=74)	5 (6.25%)
Secondary hypocorticism (*N*=75)	3 (4.05%)
Cavernous sinus invasion (*N*=76)	17 (19.8%)
Serum prolactin levels (*µ*g/L)	199 [97.6;443]

Data availability is indicated for each variable. Categorical variables are presented with counts and percentages; continuous variables are presented with median and interquartile range (IQR).

### Performance Metrics and Predictor Importance

We assess the discrimination ability of the various classifiers using two independent performance metrics: the area under the receiver operating characteristic (AUROC) and the Matthews correlation coefficient (MCC). One of the advantages of the MCC is that it is based on the full confusion matrix (i.e. true and false both positives and negatives) (48); another is that it performs well on imbalanced data sets (49). By considering the two performance indicators together we get a more detailed and comprehensive assessment of the performance of a binary classifier: whereas the AUROC indicator measures diagnostic ability by comparing the true positive rate (TPR) with the false positive rate (FPR) and varying the threshold (or cutoff) used to make the classification, the MCC is not based on varying the threshold but rather explicitly accounts for the balance ratios of the 4 entries in the confusion matrix.

The importance of each predictor is assessed within a permutation framework: as performance metric we choose the AUROC and the change in AUROC is computed when the values of a particular predictor (i.e. age) are permuted within the patients: the larger the change in the AUROC with respect to the AUROC based on the original, unpermuted data, the more important a predictor is considered to be.

### Statistical Software

All computations were performed with R version 4.0.5 (50). In particular, the machine learning workbench *mlr* (51) is used to compute and evaluate the various ML algorithms.

## Results

### Characteristics of the Study Population

Patients’ demographic and baseline characteristics are summarized in **Table 1**. For the 86 patients undergoing first-line surgery, median age was 32 years (IQR, 27 - 42 years) and 82.6% were female. A macroadenoma was diagnosed in 41 patients (47.7%). Fifty-three patients (76.8%) exhibited secondary (hypogonadotroph) hypogonadism, with secondary hypothyroidism present in 4 patients (5.3%) and secondary hypocorticism present in 3 patients (4.1%), respectively. Median prolactin levels were 199*µ*g/L (IQR, 97.6 - 443.0 *µ*g/L).

Outcomes at early and long-term follow-up are shown in **Table 2**. As for surgery alone, we noted that remission was achieved in 52 (63%) patients at early follow-up, and in 49 (59%) patients in the long-term. For the control of hyperprolactinemia, DA was ultimately required in 19 (22%) patients at early follow-up, and in 31 (36%) patients at the long-term follow-up. All of the patients with long-term DA dependency did not show remission at early follow-up.

**Table 2:** Patients’ characteristics at early (30 days postoperatively) and long-term follow-up.

Characteristics	Early Follow-up	Long-term Follow-up
BMI (kg/m^2^)	25.0 [21.4;28.7] (N=63)	25.8 [21.3;29.0] (N=73)
Secondary hypocorticism	3/75 (4.00%)	3/84 (3.57%)
Secondary hypogonadism	33/52 (63.5%)	13/48 (27.1%)
Secondary hypothyroidism	4/76 (5.26%)	8/85 (9.41%)
Serum prolactin levels (*µ*/L)	15.0 [7.33;72.8] (N=76)	12.7 [7.60;20.4] (N=83)
DAs (i.e. Cabergoline)	5/85 (5.88%)	20/85 (23.5%)
DAs (i.e. Bromocriptine)	14/85 (16.5%)	11/85 (12.9%)
Outcomes		
DA dependency [*primary*]	19/85 (22.3%)	31/85 (36.5%)
Control of hyperprolactinemia [*secondary*]	50/76 (65.8%)	76/83 (91.6%)

Data availability is indicated for each variable. Categorical variables are presented with counts and percentages; continuous variables are presented with median and interquartile range (IQR).

Thereby, daily doses of DA agonists at early follow-up were as follows (mean ± SD): bromocriptine 7.1 ± 1.0 mg, and cabergoline 0.08 ± 0.03 mg. Daily doses at last follow-up were 5.9 ± 2.9 mg for bromocriptine, and 0.09 ± 0.03 mg for cabergoline.

Patients with short-term remission had significantly lower PRL levels than those without short-term remission (133 μg/L (IQR 78–224 μg/L) vs. 303 μg/L (IQR 211–900 μg/L), p < 0.001).

Cavernous sinus invasion was a significant predictor for long-term dependence on DAs (p=0.03) when excluding the predictor remission from the multivariable regression due to the near-complete separation.

Secondary hypothyroidism was present in 8 patients (9.4%), with levothyroxine substitution therapy being prescribed in all but one of them.

Diabetes insipidus (DI) or Syndrome of inappropriate antidiuretic hormone secretion (SIADH) was biochemically documented in case of clinical suspicion only. Thereby, SIADH was present in 10%, and DI in 13% of patients, respectively.

### Hyperparameter Tuning

**Figure 1:**
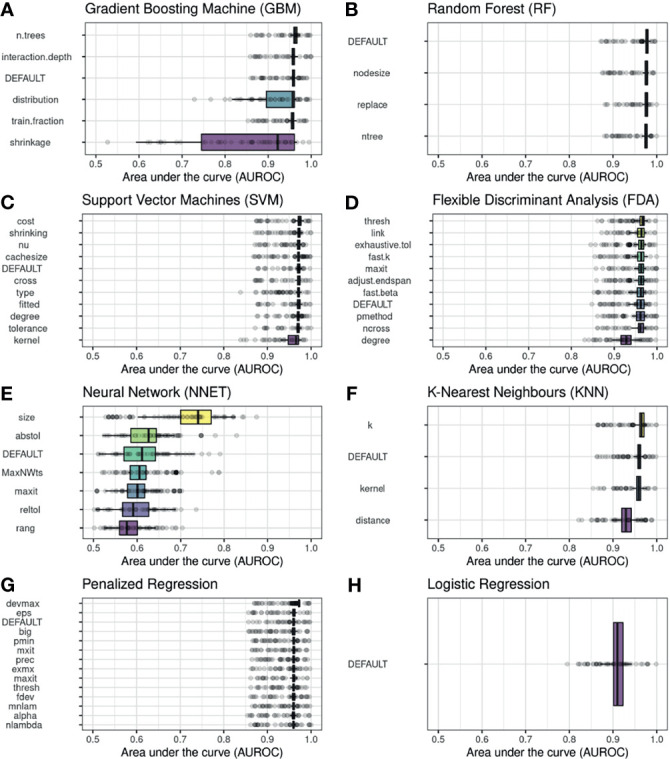
Hyperparameter tuning in our set of machine learning classifiers. The impact of varying the default values of a single hyperparameter on the area under the curve (AUROC) is illustrated for a selection of hyperparameters in each algorithm (shown on the ordinate). Each hyperparameter is sampled 50 times and its performance is assessed within a repeated cross-validation sampling (three-fold, 4-repeats), resulting in an AUROC distribution, which is illustrated with a box and whiskers plot. The outcome was dependence on dopamine agonists at long-term follow-up. For comparison, the range of AUROC values derived using the default hyperparameter settings are shown as DEFAULT in each panel. Due to the repeated cross-validation sampling, the default hyperparameter settings also feature AUROC distributions, despite using only a fixed set of hyperparameters.

The range of AUROC values derived from perturbing the default hyperparameters for each classifier is illustrated in **Figure 1**. The target variable for this hyperparameter sensitivity analysis was DA-dependency at the long-term follow-up (primary outcome). Most classifiers perform very well, with AUROC values above 0.9 with default hyperparameter settings. Only a few classifiers displayed significant sensitivity of hyperparameter settings, and thus had the potential to achieve higher AUROC performances by hyperparametertuning, notably the Gradient Boosting Machine (GBM), the Neural Network (NNET) and the k-nearest neighbor (KNN) classifiers. Note that the logistic regression features performance metrics similar to those of the other algorithms, even outperforming them in the case of the NNET classifier. From here onwards, we selected two hyperparameters for each classifier, based on their individual capability in increasing the discrimination ability of the corresponding classifier, and sampled them jointly.

### Relationship Between the Two Performance Metrics AUROC and MCC

**Figure 2:**
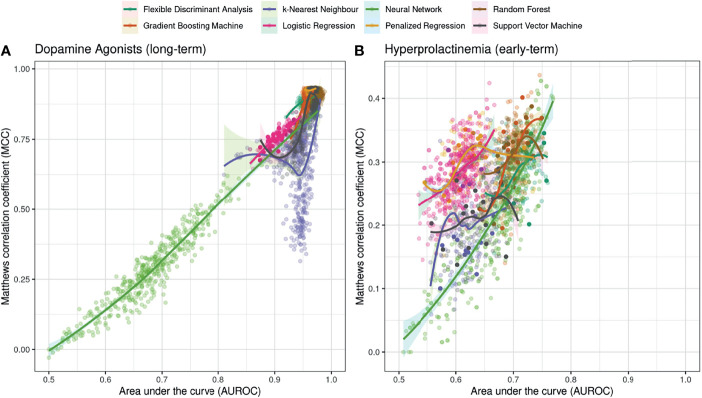
Relationship between two performance metrics in a set of supervised classification algorithms resulting from randomly sampling two hyperparameters in each algorithm (N=500 samples). The area under the curve (AUROC) performance indicator is shown on the abscissa, whereas the corresponding value for the Matthews correlation coefficient (MCC) is shown on the ordinate. The outcomes are **(A)** dependency on DA on long-term follow-up and **(B)** successful control of hyperprolactinemia at early follow-up. For illustration purposes, a Locally Weighted Scatterplot Smoothing (LOESS) curves with associated 95% confidence intervals are shown for each classification algorithm.

**Figure 2** depicts the relationship between two performance metrics in a set of 500 randomly sampled hyperparameters: the area under the curve (AUROC) on the abscissa and the Matthews correlation coefficient (MCC) on the ordinate are shown for each classifier and hyperparameter combination.

We found a quasi-linear relationship between the AUROC and the MCC for most algorithms, suggesting that a high AUROC performance for an algorithm also features a high MCC. Interestingly, some ML methods such as the k-nearest neighbor and penalized regression display non-linear relationships in AUROC and MCC, implying that some choices of hyperparameters result in performance gains only in one of the performance metrics, while the performance measured by the other metric decreases. **Figure 2** further shows that hyperparameter tuning can result in very broad performance ranges, notably by sampling the size of a neural network for the prediction of the primary outcome (**Figure 2A**). A further insight from **Figure 2** is that the range of performances of the standard logistic regression resulting from the RepCV-sampling procedure can be compared to the performance range of “modern” machine learning algorithms resulting from hyperparameter sampling.

**Figure 2** further highlights that depending on the choice of hyperparameters, the classifiers can display similar AUROC performances; however, their performance as measured with the MCC metric can be significantly different – at least for the outcomes and predictors available for the present study. For example for the prediction of successful hyperprolactinamia at early follow-up, a Neural Network with a particular choice of hyperparameters can display an AUROC of 0.65 and a (low) MCC of roughly 0.2, whereas a logistic regression can feature the same AUROC value of 0.65 but a comparatively larger MCC of 0.3 (**Figure 2B**). The added value of ML methods in the modeling setup here is the result that hyperparameter tuning provides the opportunity for some ML to outperform logistic regression in both metrics, thus constituting an added benefit with respect to the more traditional prediction by logistic regression. Note, however, that the performance of logistic regression can be considered competitive with respect to other algorithms, and hyperparameter tuning is often required to achieve the performance gain displayed by other machine learning methods.

Overall, the take-home message of this Figure is that examining the two performance indicators together provides a more comprehensive picture of the overall discrimination ability of a particular classifier, and can facilitate the comparison and choice of a particular machine learning algorithm.

### Primary and Secondary Outcomes

**Figure 3:**
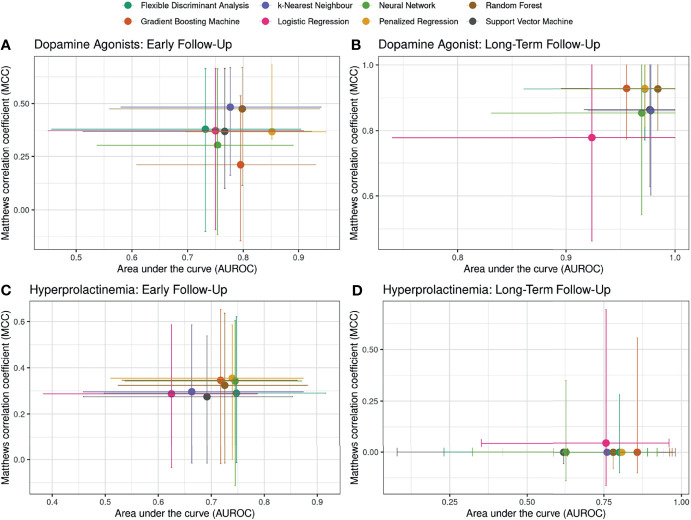
Area under the curve (AUROC) and Matthews correlation coefficient (MCC) values for the outcomes at early- and long-term follow-up. Median and 95% confidence intervals are shown, where the latter were derived in a repeated cross-validation sampling (three-fold, 100-repeats). For each machine learning algorithm, two influential hyperparameters (refer to **Figure 1**) were sampled 100 times and the hyperparameters settings resulting in the best AUROC performance were selected.

**Figure 3** shows the median AUROC and MCC values and associated 95% confidence intervals (computed from the repeated cross-validation) for early- and long-term dependency of DAs based on optimized hyperparameter settings. In terms of predicting the DA dependence, **Figure 3B** demonstrates that the prediction performance is particularly high for the long-term (primary endpoint): a Random Forest classifier features a median AUROC performance of 0.98 and a MCC of 0.93. In this case, all ML algorithms consistently outperform logistic regression. For the prediction of DA dependence on early follow-up, the classifiers feature only moderate performances (median AUROC range: 0.73–0.85, median MCC range: 0.21–0.48, **Figure 3B**).

The high prediction performance of the classifiers for the primary outcome is strongly related to the association of remission after 30 days: of the 52 out of 83 patients who did not show DA dependency, 49 did show remission at 30 days, whereas all of the patients with long-term DA dependence did not show remission after 30 days. We thus find almost complete separation in these two variables. The importance of remission at 30 days will be further quantified below.

To predict the control of hyperprolactinemia at early follow-up, all classifiers displayed only moderate performance, with median AUROC values ranging from 0.62 to 0.75 and median MCC performance ranging from 0.27 to 0.35. In terms of predicting the long-term outcome in hyperprolactinemia, the overall performance was slightly increased, with moderate median AUROC values ranging from 0.62 (Support Vector Machine) to 0.86 (Gradient Boosting Machine). All MCC values are equal to zero, likely due to the small sample size and the imbalanced datasets: an MCC of zero can result when a row or a column of the confusion matrix measures exactly zero, while the other two entries feature non-zero entries (14). As there were only seven patients with a successful long-term hyperprolactinemia outcome, the data splitting in the cross-validation might result in zero entries in the confusion matrix.

Overall, we noted that there was no single classifier outperforming all other classifiers and that different algorithms performed best for different times and different outcomes. In the context of this near-complete separation for the primary outcome and remission at 30 days, **Figure 3** indicates that the ML algorithms might be more capable of handling such variable separation compared to logistic regression, as these classifiers showed better performance metrics and narrower confidence ranges. The complete data table of **Figure 3** is provided in the **Supplementary Material**.

To complete the evaluation of the classifiers on outcomes considered in our analyses, **Table 3** presents the performance metrics for a *super learner*, which combines the predictions of individual *base learners* (see Methods). The performance of the *super learner* ranks generally high compared to most individual base learners, however the *super learner* does not always outperform individual base learners.

**Table 3:** Performance metrics of a stacked *super learner* combining the outcome predictions of the individual classifiers (referred to as *base learners*; see method section).

Outcome	AUROC	MCC	SENS	SPEC	PPV	NPV
Dopamine Agonist dependency						
Long-term	0.97 (0.92–1.00)	0.85 (0.60–1.00)	0.94 (0.83–1.00)	0.91 (0.64–1.00)	0.95 (0.82–1.00)	0.91 (0.75–1.00)
Early-term	0.80 (0.57–0.94)	0.38 (−0.08 to 0.77)	0.89 (0.73–1.00)	0.46 (0.14–0.86)	0.86 (0.77–0.95)	0.56 (0.15–1.00)
*Control of hyperprolactinemia*						
Long-term	0.80 (0.58–0.97)	0.11 (−0.12 to 0.69)	0.17 (0.00–0.67)	0.95 (0.80–1.00)	0.23 (0.00–1.00)	0.93 (0.88–0.96)
Early-term	0.69 (0.50–0.83)	0.27 (−0.02 to 0.57)	0.53 (0.22–0.78)	0.74 (0.53–0.94)	0.52 (0.33–0.76)	0.76 (0.64–0.88)

Outcomes are dependency on dopamine agonists and successful control of hyperprolactinemia at early-and long-term follow-up. Mean and 95% confidence intervals from a repeated cross-validation are shown.

AUROC, area under the receiver operating characteristic; MCC, Matthews correlation coefficient; SENS, sensitivity; SPEC, specificity; PPV, positive predictive value; NPV, negative predictive value.

### Variable Importance

**Figure 4:**
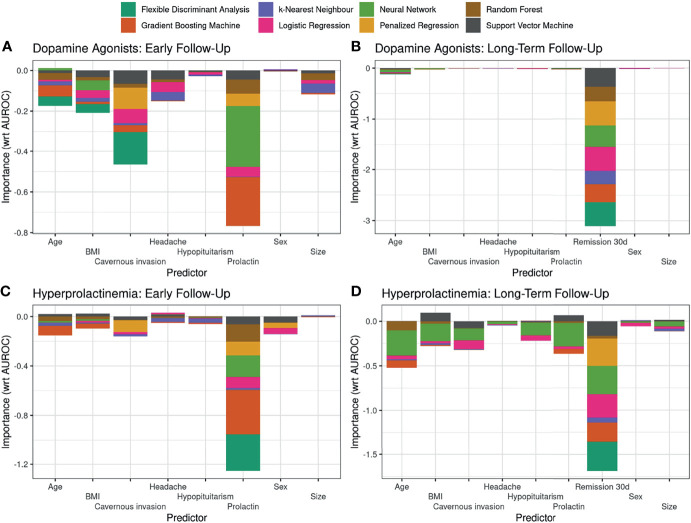
Importance of the available set of variables in predicting early and long-term outcome. The variable importance metric is based on a permutation approach, where the impact of perturbing the values of a given predictor on a particular performance metric [in this case: area under the curve (AUROC)] is assessed: the larger the decrease in the AUROC metric, the more important a predictor is considered. The variable importance is assessed for each classification algorithm with optimized hyperparameters, and the importance values for each predictor are simply stacked upon each other to illustrate the overall importance of a particular predictor and to visualize the inter-algorithm agreement in the assessment of the importance of a single predictor.

We next examined the importance of each variable in predicting the outcome at early and long-term follow-up. The decrease in the AUROC values when the values of a particular predictor are perturbed is illustrated in **Figure 4**. Thus, the more negative the importance metric on the ordinate is, the more important the predictor is considered to be. Thereby, prolactin levels are the most important predictors at early follow-up, both for the control of hyperprolactinemia and for dependence on DAs (**Figures 4A, C**). In addition, remission from hyperprolactinemia at 30 days is the most important predictor for the long-term dependency of DAs, and this finding is robust across most classifiers, likely due to near-complete separation in the two variables (**Figures 4B, D**). Of secondary importance are the presence of prolactinoma invasion into the cavernous sinus, as well as patients’ age, BMI and sex.

## Discussion

Our results highlight the benefits of employing a ML approach in addition to traditional methods such as logistic regression for outcome prediction in prolactinoma patients treated with first-line surgery, in particular in a situation of near-complete variable separation, as is the case here for the primary outcome with the predictor *remission 30 days*.

In a systematic review featuring 71 studies, no superior performance of ML algorithm compared to logistic regression was found for clinical prediction models (52). In a similar vein, it was demonstrated that logistic regression and ML methods have a similar ability to predict major chronic diseases with low incidences and only simple clinical predictors (53). Against this background, we demonstrate that there was no one-algorithm-fits-all solution in predicting early and long-term outcome in prolactinoma patients treated with first-line surgery: different algorithms performed best for different outcomes and at different times, and there are instances when logistic regression featured similar (or better) performance scores than ML methods (**Figure 3A**). We thus argue and highlight in this study that by jointly examining two independent performance metrics – the area under the receiver operating characteristic (AUROC) and the Matthews correlation coefficient (MCC) – the discrimination capacity of a set of binary classifiers can be more holistically investigated than by focusing on a single performance metric such as the AUROC. Importantly, with the stacking framework of the *super learners* (47), ML offers a viable methodology to combine different classifiers. In general, the *super learner* exhibits a high performance metric compared to individual classifiers. In this regard, ML adds to the current statistical methods when it comes to outcome prediction of first-line surgery in prolactinoma patients.

Our data indicate that baseline serum prolactin levels are by far the most important outcome predictor at early follow-up, whereas remissions at 30 days dominated the importance of long-term dependence on DAs. Initial high serum PRL levels have been associated with recurrence of hyperprolactinemia (54, 55), corroborating our results. Likewise, in a large cohort of prolactinoma patients, Mattogno and colleagues reported that in those with a follow-up of > 5 years, surgery and female gender were independent predictors of control of hyperprolactinemia (17). Just as in women symptoms such as amenorrhea are investigated at an early time-point, subsequent prolactin levels are usually not as high as in men harboring larger adenomas due to unreported or subclinical symptoms of hypogonadism (13, 56, 57).

DAs can be tapered 24 months after initiation of medical therapy in case of normalization of the respective serum PRL values (1). However, early recurrence of hyperprolactinemia has been described (58) following discontinuation of DAs, in particular in patients with macroprolactinomas (14, 59–61), or those with adenoma extension into the cavernous sinus (11). In surgical series, recurrences in as many as one-third of patients with prolactinomas have been reported, including late recurrences of more than 10 years (62). In this regard, reporting the number of patients who remain off medication is an important outcome predictor (11, 63), as surgery can be an effective alternative treatment option in selected patients (11–13, 64, 65). However, whether surgery of prolactinomas dominates DAs as a first-line approach or a second-line treatment is a matter of debate, with the PRolaCT trial hopefully providing insights on this important issue (16).

This study has inherent limitations. First, the set of available variables and study population size is somewhat limited, suggesting only exploratory findings with regard to the prediction capacity of the models (66). However, the available dataset still represents one of the largest cohorts of patients with a surgery-first approach, reaching a long-term follow-up of almost 10 years, which we think is crucial. In addition, the dataset features missing data in variables, and the (single) imputation approach in the repeated cross-validation might impact the training and test sets and thus the two performance metrics. Second, we consider only a limited set of ML classifiers. Third, computational resources constrained the sampling of the hyperparameter space of each classifier. However, given the robustness of the classifier performance – i.e., consider the similar AUROC and MCC performances in **Figure 3** – it seems not very likely that sampling more hyperparameters would have resulted in a fundamental performance increase.

From a clinical point of view, a follow-up period of <24 months in a few patients may have confounded the results of long-term DAs dependence, as our treatment strategy follows current consensus guidelines in tapering DAs 24 months after initiation of the medical therapy in case of normalized serum prolactin levels and/or prolactinoma size reduction of >50%. Thereby, not all patients were subsequently screened with a pituitary MR in case of normoprolactinemia at follow-up. In addition, we cannot exclude that a very small number of prolactinomas diagnosed as prolactinoma were GH co-secreting adenomas or non-secreting adenomas. Finally, not all patients were systematically screened for growth hormone deficiency using validated dynamic testing if there was not a clinical suspicion for significant adult GH-deficiency, and the agreement of the patients to treat the condition by daily injections.

## Conclusion

There were benefits in employing a ML approach and of using a set of diverse classification algorithms to predict long-term DA-dependency following first-line surgery in prolactinoma patients. We can confirm that baseline prolactin levels are by far the most important outcome predictor at early follow-up, whereas remission at 30 days dominates the prediction skill for DA- dependence over the long-term.

## Data Availability Statement

The raw data supporting the conclusions of this article will be made available by the authors, without undue reservation.

## Ethics Statement

The Cantonal Ethikkommission KEK Bern (Bern, Switzerland) approved the project (KEK no 10-10-2006 and 8-11-2006). Written informed consent for participation was not required for this study in accordance with the national legislation and the institutional requirements.

## Author Contributions

Conception and design: LA, EC, MH. Acquisition of data: JF, LA. Analysis and interpretation of data: MH, LA. Drafting the article: MH, LA. Critically revising the article: MH, ML, EC, LA. Reviewed submitted version of manuscript: all authors. Statistical analysis: MH. Administrative/technical/material support: ML. Study supervision: LA, EC. All authors contributed to the article and approved the submitted version.

## Conflict of Interest

The authors declare that the research was conducted in the absence of any commercial or financial relationships that could be construed as a potential conflict of interest.

## Publisher’s Note

All claims expressed in this article are solely those of the authors and do not necessarily represent those of their affiliated organizations, or those of the publisher, the editors and the reviewers. Any product that may be evaluated in this article, or claim that may be made by its manufacturer, is not guaranteed or endorsed by the publisher.

## Abbreviations

DA, dopamine agonist; MCC, Matthews correlation coefficient; ML, machine learning; MRI, magnetic resonance imaging; PRL, prolactin; TSS, transsphenoidal surgery.
