# Supplementary material for: Machine Learning for Outcome Prediction in First-Line Surgery of Prolactinomas
Source: Front Endocrinol (Lausanne). 2022 Feb 16;13:810219. doi: 10.3389/fendo.2022.810219 (PMC8888454; doi:10.3389/fendo.2022.810219)
Supplement: Supplementary file 1 [file Table_1.docx]

**Supplementary Material**

**Table SM 1**: Performance metrics as depicted in Figure 3. Mean and 95% confidence intervals from a repetead cross-validation are shown for each classifier.

| **AUROC** | **MCC** | **Method** |
| --- | --- | --- |
| *Dependency on DAs in the long-term* | | |
| 0.98 (0.92 - 1.00) | 0.93 (0.80 - 1.00) | Random Forest |
| 0.98 (0.92 - 1.00) | 0.86 (0.60 - 1.00) | k-Nearest Neighbour |
| 0.98 (0.92 - 1.00) | 0.86 (0.63 - 1.00) | Support Vector Machine |
| 0.97 (0.86 - 1.00) | 0.93 (0.77 - 1.00) | Flexible Discriminant Analysis |
| 0.97 (0.92 - 1.00) | 0.93 (0.81 - 1.00) | Penalized Regression |
| 0.97 (0.83 - 1.00) | 0.85 (0.54 - 1.00) | Neural Network |
| 0.96 (0.90 - 1.00) | 0.93 (0.77 - 1.00) | Gradient Boosting Machine |
| 0.92 (0.74 - 1.00) | 0.78 (0.46 - 1.00) | Logistic Regression |
| *Dependency on DAs at early follow-up* | | |
| 0.85 (0.70 - 0.95) | 0.37 ( 0.34 - 0.68) | Penalized Regression |
| 0.80 (0.56 - 0.94) | 0.48 ( 0.12 - 0.67) | Random Forest |
| 0.80 (0.61 - 0.93) | 0.21 (-0.14 - 0.54) | Gradient Boosting Machine |
| 0.78 (0.58 - 0.94) | 0.48 ( 0.16 - 0.67) | k-Nearest Neighbour |
| 0.77 (0.51 - 0.92) | 0.37 ( 0.10 - 0.67) | Support Vector Machine |
| 0.75 (0.54 - 0.89) | 0.31 (-0.11 - 0.66) | Neural Network |
| 0.75 (0.45 - 0.91) | 0.37 (-0.09 - 0.67) | Logistic Regression |
| 0.73 (0.46 - 0.90) | 0.38 (-0.10 - 0.66) | Flexible Discriminant Analysis |
| *Control of hyperprolactinemia in the long-term* | | |
| 0.86 (0.63 - 0.97) | 0.00 (-0.10 - 0.56) | Gradient Boosting Machine |
| 0.81 (0.42 - 0.89) | 0.00 ( 0.00 - 0.00) | Penalized Regression |
| 0.80 (0.23 - 0.89) | 0.00 (-0.10 - 0.28) | Flexible Discriminant Analysis |
| 0.78 (0.59 - 0.96) | 0.00 (-0.08 - 0.00) | Random Forest |
| 0.76 (0.42 - 0.94) | 0.00 ( 0.00 - 0.00) | k-Nearest Neighbour |
| 0.76 (0.35 - 0.96) | 0.04 (-0.16 - 0.69) | Logistic Regression |
| 0.63 (0.32 - 0.92) | 0.00 (-0.14 - 0.35) | Neural Network |
| 0.62 (0.08 - 0.98) | 0.00 (-0.06 - 0.00) | Support Vector Machine |
| *Control of hyperprolactinemia at early follow-up* | | |
| 0.75 (0.50 - 0.92) | 0.29 (-0.01 - 0.62) | Flexible Discriminant Analysis |
| 0.75 (0.54 - 0.87) | 0.34 (-0.11 - 0.60) | Neural Network |
| 0.74 (0.51 - 0.88) | 0.35 ( 0.00 - 0.59) | Penalized Regression |
| 0.73 (0.52 - 0.88) | 0.32 (-0.02 - 0.64) | Random Forest |
| 0.72 (0.53 - 0.86) | 0.35 (-0.02 - 0.65) | Gradient Boosting Machine |
| 0.69 (0.46 - 0.85) | 0.27 (-0.02 - 0.54) | Support Vector Machine |
| 0.66 (0.46 - 0.88) | 0.30 (-0.01 - 0.59) | k-Nearest Neighbour |
| 0.62 (0.38 - 0.79) | 0.29 (-0.03 - 0.59) | Logistic Regression |
